# Plasma NOV/CCN3 Levels Are Closely Associated with Obesity in Patients with Metabolic Disorders

**DOI:** 10.1371/journal.pone.0066788

**Published:** 2013-06-13

**Authors:** Jihane Pakradouni, Wilfried Le Goff, Claire Calmel, Bénédicte Antoine, Elise Villard, Eric Frisdal, Marianne Abifadel, Joan Tordjman, Christine Poitou, Dominique Bonnefont-Rousselot, Randa Bittar, Eric Bruckert, Karine Clément, Bruno Fève, Cécile Martinerie, Maryse Guérin

**Affiliations:** 1 INSERM, UMR_S938, Saint-Antoine Research Center, Saint-Antoine Hospital, Paris, France; 2 Pierre and Marie Curie University–Paris 6, UMR_S938, Paris, France; 3 Sisène SAS, Paris Santé Cochin Incubator, Paris, France; 4 INSERM, UMR_S939, Pitié-Salpêtrière Hospital, Paris, France; 5 INSERM, U872, Nutriomic team 7, Cordelier Research Center, Paris, France, Pierre et Marie Curie University–Paris 6, Paris, AP-HP, Human Nutrition Research Center (CRNH), Pitié-Salpêtrière Hospital, Paris, France; 6 Pierre and Marie Curie University –Paris 6, UMR_S939, Paris, France; 7 Department of Endocrinology, AP-HP, Pitié-Salpêtrière Hospital, Paris, France; 8 Metabolic Biochemistry Department, AP-HP, Pitié-Salpêtrière Hospital, Paris, France; 9 Cardiometabolism and Nutrition Institute, ICAN, Paris, France; 10 INSERM, UMR_S698, Bichat-Claude Bernard Hospital, Paris, France; 11 Pharmacy Faculty, Saint Joseph University, Beirut, Lebanon; Clermont Université, France

## Abstract

**Objective:**

Evidence points to a founder of the multifunctional CCN family, NOV/CCN3, as a circulating molecule involved in cardiac development, vascular homeostasis and inflammation. No data are available on the relationship between plasma NOV/CCN3 levels and cardiovascular risk factors in humans. This study investigated the possible relationship between plasma NOV levels and cardiovascular risk factors in humans.

**Methods:**

NOV levels were measured in the plasma from 594 adults with a hyperlipidemia history and/or with lipid-lowering therapy and/or a body mass index (BMI) >30 kg/m^2^. Correlations were measured between NOV plasma levels and various parameters, including BMI, fat mass, and plasma triglycerides, cholesterol, glucose, and C-reactive protein. NOV expression was also evaluated in adipose tissue from obese patients and rodents and in primary cultures of adipocytes and macrophages.

**Results:**

After full multivariate adjustment, we detected a strong positive correlation between plasma NOV and BMI (r = 0.36 p<0.0001) and fat mass (r = 0.33 p<0.0005). According to quintiles, this relationship appeared to be linear. NOV levels were also positively correlated with C-reactive protein but not with total cholesterol, LDL-C or blood glucose. In patients with drastic weight loss induced by Roux-en-Y bariatric surgery, circulating NOV levels decreased by 28% (p<0.02) and 48% (p<0.0001) after 3 and 6 months, respectively, following surgery. In adipose tissue from obese patients, and in human primary cultures NOV protein was detected in adipocytes and macrophages. In mice fed a high fat diet NOV plasma levels and its expression in adipose tissue were also significantly increased compared to controls fed a standard diet.

**Conclusion:**

Our results strongly suggest that in obese humans and mice plasma NOV levels positively correlated with NOV expression in adipose tissue, and support a possible contribution of NOV to obesity-related inflammation.

## Introduction

NOV/CCN3 [1] is a founder of the CCN (Cyr61/CCN1, CTGF/CCN2, NOV/CCN3) family of multitasking matricial proteins which regulate several cellular functions, such as migration, proliferation, differentiation, survival, apoptosis and extracellular matrix remodelling in a cell-specific manner[2]. *In vivo*, CCN proteins are key players in organogenesis, inflammation [3], injury repair, fibrotic diseases and cancer [2].

NOV is a circulating protein that is also detected in diverse human tissues including the adrenal cortex, kidney, musculoskeletal and central nervous systems, heart and blood vessels [4]–[7]. Several reports have shown NOV expression altered in tumors derived from many of these tissues [5], [8]–[11] and quantification of NOV in human adrenocortical tumors [5], renal carcinomas [12], melanomas [13] and Ewing's sarcoma [14] has defined NOV as a potential prognosis biomarker.

In 2003, our group developed the first enzyme immunoassay specific to human NOV allowing the detection of NOV protein in biological fluids, such as serum, amniotic fluid and cerebrospinal fluid [7]. However, no prospective or retrospective study of its plasma concentration in physiological or pathological situations has been reported yet, especially in diseases other than cancer.

Recent reports have shown the importance of NOV during cardiac development and vascular homeostasis [15]–[17]. Several observations have strongly suggested that NOV is modulated by cytokines in several systems [3], [18], [19] and regulates the expression of chemokines, such as CCL-2 and CXCL-1 [20]. Because these inflammation-related genes are also crucial contributors to the inflammatory component of atherosclerosis [21], [22], a modulation of NOV concentration in this pathophysiological context could be important.

This study investigated circulating NOV changes in a cohort of adult patients recruited to screen cardiovascular-related diseases and to explore a possible relationship between NOV and established cardiovascular disease risk parameters. We provide evidence that a strong correlation exists between plasma NOV levels, BMI and fat mass. In addition, we show that NOV is present in adipose tissue, and is expressed by adipocytes and macrophages. Collectively, these findings strongly suggest that NOV could play a role in the development of obesity.

## Research Design and Methods

### Study population

A total of 594 individuals (242 men and 352 women), older than 18 years, were recruited for this study. The patients addressed at our out-patient clinic for Dyslipidemia and the Prevention of Cardiovascular Disease (Pitié-Salpêtrière Hospital, Paris, France) had a history of hyperlipidemia (total cholesterol ≥250 mg/dl and/or LDL-cholesterol (LDL-C) ≥160 mg/dl and/or triglycerides ≥150 mg/dl) and/or were on lipid-lowering therapy. Furthermore, patients at our out-patient clinic for Human Nutrition (Pitié-Salpêtrière Hospital, Paris, France) had a body mass index (BMI) ≥30 kg/m^2^. Cigarette smoking and physical activity was evaluated as binary variables: currently smoking or non-smoking and sedentary or engaged in regular physical exercise (defined as at least 30 minutes of exercise per day). Fifteen percent of the subjects were current smokers. Patients were separated into three groups with respect to their alcohol consumption: abstention or low alcohol intake (<10 g/day), moderate consumption (10–30 g/day) and elevated consumption (>30 g/day). Height and weight were measured with subjects wearing light clothing and without shoes. Patients were classified as normal weight (BMI <25 kg/m^2^), overweight (25 kg/m^2^<BMI<30 kg/m^2^) or obese (BMI>30 kg/m^2^). Approximately 60% of the participants were obese with a BMI>30 kg/m^2^ (70% of the women and 45% of the men). Fat mass was measured using dual energy x-ray absorptiometry (DXA). Among the obese patients, 15 were followed before and 3 and 6 months after gastric bypass as described previously [23].

Approximately 12% of subjects were hypercholesterolemic with plasma total cholesterol levels above 250 mg/dl and/or a LDL-C above 160 mg/dl (12.5% of women and 11.6% of men). Approximately 35% of patients were receiving a lipid-lowering drug. Overall, 48% of the men and 26% of the women were taking a lipid-lowering therapy. Approximately 88% of patients receiving a lipid-lowering therapy, 87% of men or 90% of women, were treated with a statin in mono-therapy (87%) or bi-therapy (13%). In addition, approximately 11% of subjects displayed a fasting blood glucose >7 mmol/l (9% of women and 14% of men). Finally, approximately 40% of them corresponded to post-menopausal women and approximately 30% of women were receiving a contraceptive treatment, or estrogen replacement therapy.

Blood samples obtained from subjects after a 12-hour overnight fast were collected into sterile EDTA-containing tubes (final concentration 1 mg/ml). Plasma was separated from blood cells by centrifugation at 2500 rpm for 20 minutes at 4°C. The study was performed in accordance with the ethical principles set forth in the Helsinki Declaration and the clinical investigation was approved by the Ethics Committee of Pitié-Salpêtrière Hospital (Paris, France). Written informed consent was obtained from all subjects.

### Animals

All experimental procedures on mice were conducted in accordance to the guidelines of the Charles Darwin's Ethics Committee for animal experiments (Ce5/2010/034). Three months-old male C57/Bl6 mice were fed ad libitum either with a high-fat diet (HFD), (40% kcal fat and 40% kcal carbohydrates,TD96132, Harlan) or a standard diet (SD) for 12 weeks. At the end of the experiment, after a 6 h-fast, mice were anesthetized and euthanized by cervical dislocation, blood was collected in EDTA tubes and rapidly centrifuged to prepare plasma, and adipose tissue were removed, weighted and immediately frozen in liquid nitrogen and stored at −80°C for RNA extraction.

### Biochemical Measurements

Plasma lipid and glucose levels were assayed by standardized techniques in an autoanalyzer (Konelab 20) and by using kits from Roche Diagnostics for total cholesterol and from ThermoElectron (Courtabeuf, France) for triglycerides (TG) and glucose. HDL-cholesterol (HDL-C) levels were determined by a direct method (ThermoElectron). HbA1c was determined using commercial kit from Diasys (Condom, France). Fasting plasma LDL-C was calculated using the Friedewald formula. Plasma levels of apoAI and apoB were quantified with commercial kits (ThermoElectron). The level of systemic inflammation was assessed as the plasma concentration of high sensitivity C-reactive protein (CRP) measured by a latex-enhanced immunonephelometric assay on a BN II analyser. Plasma and cell-conditioned medium NOV concentrations were measured by immunoenzymatic assays (R and D systems, Lille, France) either specific for human or mouse and validated for plasma, in our laboratory. Samples were assayed in duplicate, NOV concentration was determined by using serial dilutions of a standard recombinant human or mouse NOV and four internal controls were used to determine the inter-assay coefficients of variation.

### Immunohistochemistry

Paraffin-embedded sections (5 µm) were performed on subcutaneous and omental adipose tissue from 3 obese patients. Serial sections were stained with NOV-specific K19M antibody at a 1∶50 dilution as previously described [5], anti-CD68 at a 1∶300 dilution (Dako Corp, Trappes, France) and anti-perilipin at a 1∶100 dilution (Progen, Heidelberg, Germany). Bound antibodies were detected using biotinylated secondary antibodies followed by streptavidin horseradish peroxidase (Biocare Medical, Les Ulis, France). The peroxidase reaction was developed for 5 min in Aqeous mount AEC type (Dako, Trappes, France). The sections were counterstained with Mayer's hematoxylin solution. The flow-through (FT) resulting from K19M antibody purification and containing IgG depleted from NOV-specific IgG was used as a negative control [20].

### Preparation of human monocyte – derived macrophage and adipocyte cultures

Plasma mononuclear were cells isolated from human blood and their differentiation into macrophages was performed as previously described [24]. Human pre-adipocytes purchased from Promocell, (Heidelberg, Germany) were cultured and differentiated to mature adipocytes as recommended by the manufacturer. Differentiation efficiency was monitored by testing the induction of adipocyte marker mRNA levels, such as adiponectin, leptin and PPARgamma.

### RNA extraction, reverse-transcription and quantitative-PCR

Total RNA was extracted from mouse adipose tissue or from human primary cultures using the RNeasy total RNA minikit (Qiagen). Reverse transcription and semi-quantitative real-time PCR amplification on the ABI 7300 apparatus (Applied Biosystems, Life Technology, Courtaboeuf, France) were performed as previously described [25]. Specific primers (Proligo, Sigma-Aldrich, Lyon, France) used for amplification of target genes were designed using the Primer3 Input program and were the following: Forward 5′CTGCATTGAACAGACCACAGA3′ and reverse 5′TCTTGAACTGCAGGTGGATG3′ primers for human NOV and forward 5′CTGCATTGAACAGACCACAGA3′ and reverse 5′TCTTGAACTGCAGGTGGATG3′ for mouse NOV. The comparative Ct method [26] was used to calculate gene expression values. The ribosomal 36B4 forward 5′GATTGGCTACCCAACTGTTG3′ and reverse 5′CAGGGGCAGCAGCCACAAA3′ and S26 forward 5′AAGTTTGTCATTCGGAACATTG3′ and reverse 5′GATCGATTCCTAACAACCTTGC3′ were used as housekeeping genes for human and mouse respectively.

### Statistical analyses

Numerical variables were presented as the means ± standard deviation. Data with skewed distributions (NOV, triglyceride levels, CRP and HbA1c) were equally summarized as the median and Q1–Q3 intervals and log-transformed before further analyses. Comparisons between the means of the two groups were performed using an unpaired Student's *t*-test. The relationships between plasma NOV levels and categorical variables (alcohol consumption, smoking status and physical activity) were evaluated using ANOVA. Pearson's correlation coefficients were computed to assess the associations between continuous variables and plasma NOV levels. These analyses were equally performed by computing partial correlation coefficients adjusted for age and sex. Plasma NOV levels were modelled as a binary variable (dichotomized at the median) in primary analyses. Additional analyses were performed modelling plasma NOV levels as a continuous variable. All variables that were significantly correlated with plasma NOV concentrations in the univariate analysis were included in stepwise multiple regression models. Each explicative variable without significant independent impact on plasma NOV levels were deleted step by step. Analysis was finalized when the regression model was exclusively composed of explicative variable displaying a significant and independent impact on plasma NOV levels. Three distinct models were obtained using plasma NOV levels as the dependent variable and fat mass, physical activity, smoking and sex in the first model, fat mass, physical activity, smoking and logTG in the second model and BMI, physical exercise and smoking in the third model. Experimental data were analyzed using the SAS Package software (SAS/STAT User's Guide, Version 8 by SAS Institute Inc., Cary, NC, 1999). The R statistical software computer program version 2.13.1 was used for univariate and multivariate regression analyses. The results were considered to be statistically significant at p<0.05.

## Results

### Distribution of plasma NOV levels among the study population

The clinical and biological characteristics of the 594 patients (242 men and 352 women) enrolled in this study are presented in **Table 1**. The men were significantly older (+8.5%; p = 0.0004) than the women. The frequency of diabetes with fasting blood glucose >7 mmol/l was 12% in the overall study population. On average, plasma lipid levels were within the normal range, with women displaying a significantly higher plasma HDL-C level and lower plasma TG level than men.

**Table 1:** Clinical and biological characteristics of the study population.

	Total population (n = 594)	Men (n = 242)	Women (n = 352)
	Mean ±SD (Median/Q1–Q3)	Mean ±SD (Median/Q1–Q3)	Mean ±SD (Median/Q1–Q3)
Age (y)	50.0±15.1	52.7±14.6	48.2±15.1*
Body Mass Index (kg/m^2^)	34.1±9.5	31.4±8.3	36.0±9.8*
Fat Mass (%)	34.2±10.8	26.3±8.9	39.5±8.5*
BG (mmol/l)	5.68±1.64	5.98±1.84	5.47±1.44*
HbA1c (%)	6.10±1.02 (5.8/5.5–6.3)	6.30±1.20 (5.9/5.6–6.6)	5.98±0.85 (5.8/5.5–6.3)*
CRP (mg/l)	3.29±4.73 (1.4/0.7–4)	2.81±4.27 (1.4/0.7–3.5)	3.79±5.14 (1.5/0.6–4.7)
TC (mg/dl)	192.5±40.1	188.6±41.3	195.3±39.0
HDL-C (mg/dl)	49.2±16.6	42.0±12.2	54.2±17.4*
LDL-C (mg/dl)	117.4±35.8	115.7±37.7	118.5±34.3
TG (mg/dl)	135.1±107.7 (108/78–203)	161.7±118.4 (125.5/87–199)	116.8±94.2 (96/65–143) *
apoAI (mg/dl)	140.4±28.2	132.0±23.6	147.0±29.8*
apoB (mg/dl)	98.5±24.9	99.7±25.1	97.5±24.8
NOV (ng/ml)	6.09±3.90 (5.12/3.89–6.86)	5.41±2.33 (4.81/3.82–6.32)	6.56±4.62 (5.2/3.98–7.29)*

BMI, body mass index; BG, blood glucose; CRP, C-reactive protein; TC, total cholesterol; HDL, high-density lipoprotein; LDL, low-density lipoprotein; TG, triglycerides; apo, apolipoprotein.

* p<0.05 *versus* men.

**Figure 1:**
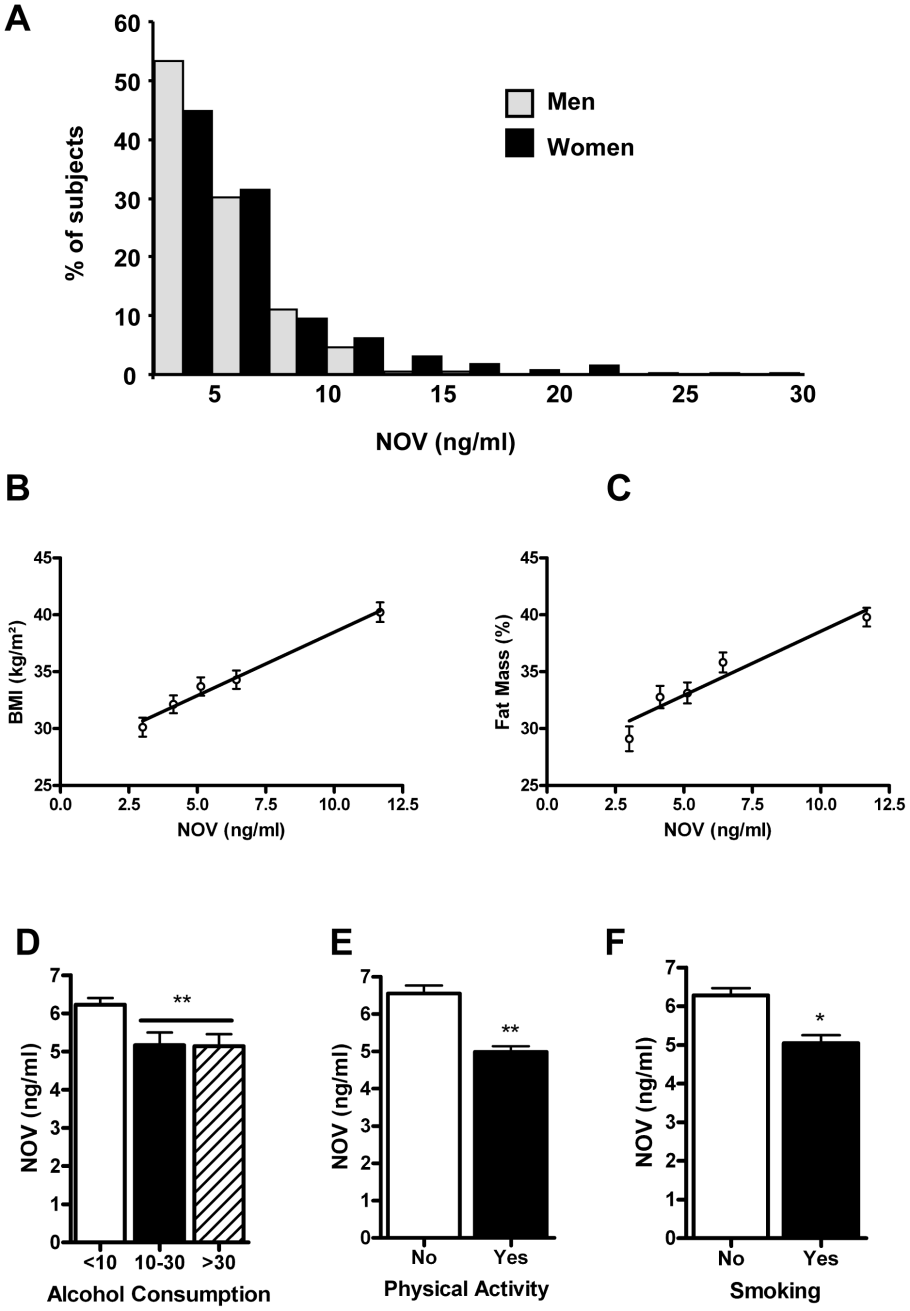
Sex, BMI, fat mass, alcohol consumption and physical exercise influence plasma NOV concentrations. A/Plasma NOV (ng/ml) distribution among 242 men (light gray bars) compared to 352 women (black bars) shows a shift toward higher values in women. The number of subjects is presented as a percentage of total men or women in the study population. B and C/Represented here are the means of body mass index (BMI kg/m^2^) (B) and the fat mass (%) (C), according to quintiles of plasma NOV concentrations. D, E and F/NOV plasma variation as a function of modifiable risk factors.(D) Alcohol consumption defined as abstention or low alcohol intake (<10 g/day), moderate consumption (10–30 g/day) and elevated consumption (>30 g/day); (E) Physical activity; (F) Smoking. Values are the means ± standard deviation (*p = 0.005; and **p<0.0001).

The average plasma NOV concentration appeared to be significantly (p<0.0001) elevated in women compared to men (**Table 1**). The median values were 4.81 ng/ml and 5.20 ng/ml in men and women respectively. In accordance with the difference in mean NOV levels between the two populations, the distribution of plasma NOV concentrations in women was shifted toward higher values compared to men (**Figure 1A**). Thus, mean levels of various clinical and biological characteristics are presented separately for men and women with circulating NOV levels dichotomized at the median (**Table 2**). Men or women with a plasma NOV concentration above the median displayed significantly (p<0.0001) higher BMI and fat mass. In addition, men displaying a plasma NOV level above the median value displayed significantly (p<0.014) higher triglyceride levels and higher CRP levels (p<0.003). Age, blood glucose, HbA1c, total cholesterol (TC), HDL-C and LDL-C were not significantly different between the subgroups of both men and women. Using a threshold value corresponding to Q3 for NOV values of 6.32 ng/ml for men and 7.29 ng/ml for women, the prevalence of elevated NOV concentration was markedly increased in hypertriglyceridemic (TG>150 mg/dl) men (46%) and in obese women (90%) identified by a BMI≥30 kg/m^2^.

**Table 2:** Clinical and metabolic characteristics of subjects according to plasma NOV levels.

	Men (Median = 4.81 ng/ml)		Women (Median = 5.20 ng/ml)	
	<Median (n = 121)	>Median (n = 121)	<Median (n = 176)	>Median (n = 176)
Age (y)	51.8±126	53.6±16.3	49.3±13.5	47.1±16.5
BMI(kg/m^2^)	29.2±7.2	33.7±8.8***	34.3±9.5	38.3±9.6***
Fat Mass (%)	24.0±8.9	28.6±8.4***	38.1±8.9	41.6±7.1***
BG (mmol/l)	5.80±1.40	6.17±2.19	5.41±1.51	5.45±1.51
HbA1c (%)	6.15±1.02 (5.8/5.6–6.4)	6.43±1.32 (6.1/5.7–6.7)	5.88±0.77(5.7/5.4–6.1)	6.06±0.93 (5.8/5.5–6.3)
CRP (mg/l)	2.01±3.21 (1.0/0.5–2.2)	3.96±5.20**(2.2/1.2–4.0)	3.87±6.65(1.3/0.6–4.0)	3.91±4.42 (2.0 0.9–5.75)
TC (mg/dl)	189.5±41.6	187.7±41.2	201.4±38.6	193.0±37.8
HDL-C(mg/dl)	43.4±13.2	40.6±11.0	53.0±15.5	54.3±16.6
LDL-C(mg/dl)	117.9±38.0	113.5±37.6	125.1±35.4	115.1±31.8
TG (mg/dl)	145.5±106.6(117/81–172)	177.9±127.6*(148/93–228)	116.6±64.0(90.5/61–145.5)	120.9±101.7(104/69.8–139.3)
apoAI (mg/dl)	132.2±26.2	131.8±20.7	148.6±28.4	144.6±30.8
apoB (mg/dl)	99.5±26.1	100.0±24.2	100.7±24.9	96.9±24.2

Data are expressed as the mean ± standard deviation. Values in parentheses indicate median/Q1–Q3. BMI, body mass index; BG, blood glucose; CRP, C-reactive protein; TC, total cholesterol; HDL, high-density lipoprotein; LDL, low-density lipoprotein; TG, triglycerides; apo, apolipoprotein.

*** p<0.0001;

** p<0.003 and *p<0.02.

### Relationship between plasma NOV concentration and cardiometabolic risk factors

We analyzed the correlations of NOV plasma levels with BMI, fat mass, blood glucose, HbA1c, C-reactive protein (CRP), TG, total cholesterol (TC), HDL-C, LDL-C, alcohol and smoking.

The analysis showed that age was inversely correlated with plasma NOV (p<0.05); however such an association disappeared after adjustment for sex or for lipid-lowering therapy (**Table 3**). BMI and fat mass as well as CRP and HbA1c positively correlated with a circulating NOV level. These strong associations remained similar after adjusting for age, sex and lipid-lowering therapy. Interestingly, the analyses of BMI or fat mass means, according to quintiles of plasma NOV concentrations, revealed that the relationships between NOV and BMI or fat mass appeared to be linear (**Figure 1** **B**–**C**). In contrast, no significant correlation was found between plasma NOV concentration and total cholesterol, HDL-C, LDL-C or blood glucose either before or after adjusting for age, sex and lipid-lowering-therapy (**Table 3**).

**Table 3:** Pearson's correlation and partial correlation coefficients with plasma NOV concentrations in the study population.

	r unadjusted	r adjusted for age	r adjusted for sex	r adjusted for lipid-lowering drugs
Age	−0.080*	-	−0.063	−0.040
BMI	0.357**	0.351**	0.339**	0.346**
Fat Mass	0.330**	0.321**	0.309**	0.317**
Log TG	0.127	0.075	0.099*	0.077
TC	−0.004	−0.002	−0.014	−0.015
LDL-C	−0.036	−0.040	−0.041	−0.053
HDL-C	0.005	0.010	−0.044	0.003
BG	0.014	0.037	0.034	0.041
Log HbA1c	0.111*	0.121*	0.106*	0.120*
Log CRP	0.212**	0.209**	0.237**	0.239**

Results are given for the overall population and for the subgroup of subjects not taking lipid-lowering medications. BMI, body mass index; BG, blood glucose; TC, total cholesterol; HDL, high-density lipoprotein; LDL, low-density lipoprotein; TG, triglycerides, CRP, C-reactive protein.

** p<0.0001 and *p<0.05.

Alcohol consumption was associated to lower (−17%; p<0.0001) plasma NOV levels compared to abstinent or low consumption (**Figure 1D**). By contrast, regular physical exercise significantly reduced plasma NOV compared to sedentary subjects (−24%; p<0.0001, **Figure 1E**). Equally, smoking was significantly associated with plasma NOV concentrations with a reduction by 19.7% (p = 0.005) of circulating NOV levels in smokers as compared to non-smokers (**Figure 1F**).

**Figure 2:**
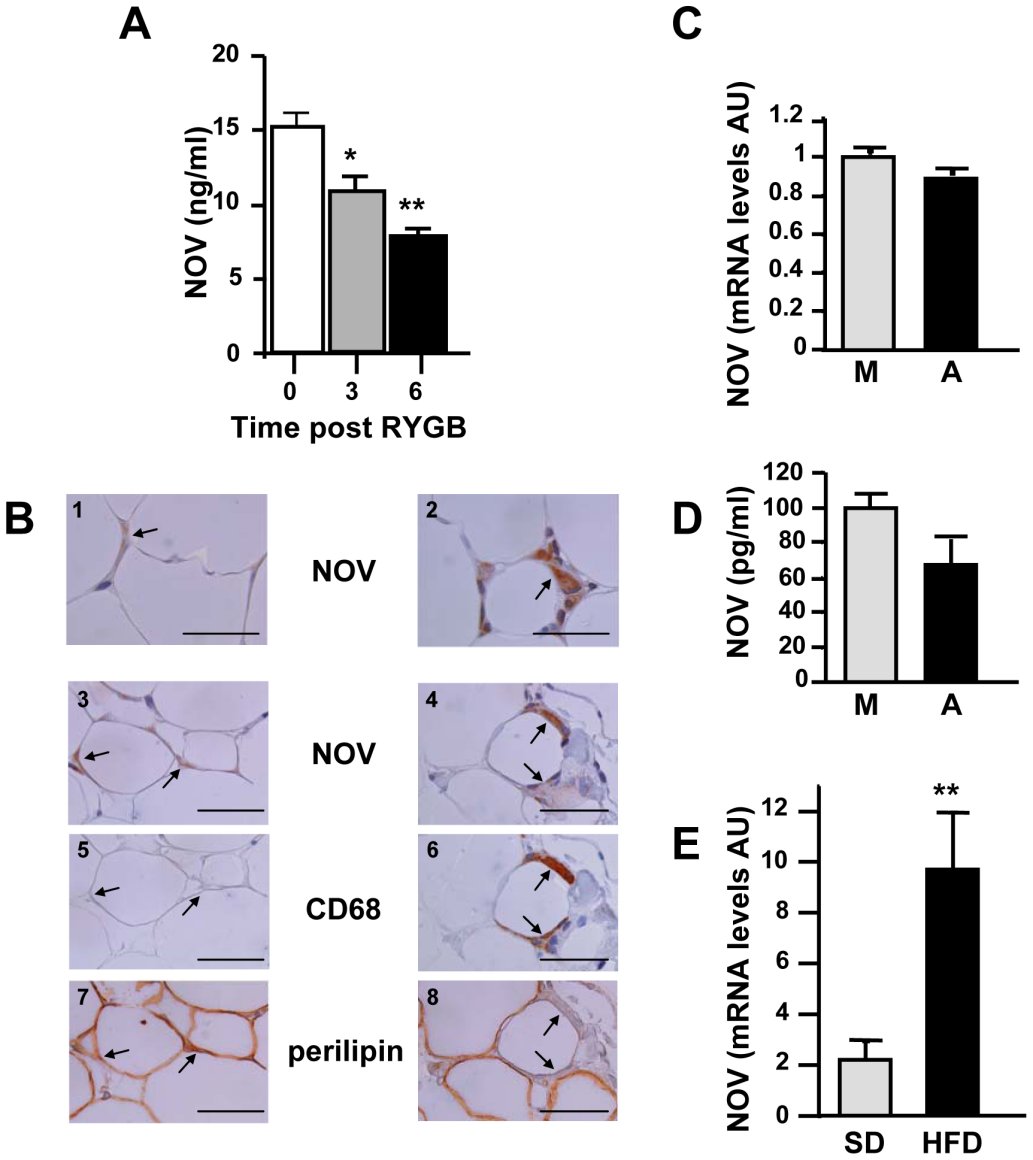
Relationship between NOV and adipose tissue. A/Time-dependent changes in plasma NOV in 15 morbidly obese women before (0) Roux-en-Y bypass (RYGB) and 3 and 6 months after surgery (*p<0.05 and **p<0.01, 3 and 6 *vs.* 0). B/Immunohistochemistry performed in the subcutaneous (1 and 2) and omental (3–8) adipose tissue of a single obese patient. The image is representative of what was observed in 3 obese subjects. The arrows indicate positive NOV staining in adipocytes (1 and 3) and macrophages (2 and 4). In serial sections corresponding to 3 and 4, respectively, the arrows indicate CD68 staining, negative in 5 and positive in 6, and the perilipin staining, positive in 7 and negative in 8. Bar  = 50 µm. C and D/NOV mRNA and protein levels in human macrophages (M) and adipocytes (A) primary cultures (AU: arbitrary units). The NOV protein concentration in 24 h-conditioned-medium was normalized for each type of cells to total RNA content. E/NOV mRNA levels were quantified by RT-qPCR in visceral adipose tissue from mice fed with a standard diet (SD) and a high fat diet (HFD) (n = 7–10/group). (AU: arbitrary units); ** P<0.01.

Multivariate analyses were performed by including all of the parameters identified above in association with plasma NOV concentration in a univariate analysis (**Table 4**). In the first model used, a multiple linear regression analysis using plasma NOV concentrations as the dependent variable showed that fat mass, physical exercise, alcohol consumption, smoking and sex in a lesser extent were independent predictors of circulating NOV levels. In the second model used, we observed fat mass, physical activity, alcohol consumption, smoking, and TG levels as independent determinants of plasma NOV concentrations. In the third model used, we identified BMI, physical exercise, alcohol consumption and smoking as independent determinants of plasma NOV concentrations. Taken together, multivariate analyses revealed that fat mass, BMI, physical activity, alcohol consumption, smoking, gender, and plasma TG levels were significantly and independently associated with plasma NOV concentrations. A stepwise analysis showed that, of the total variance of plasma NOV concentration, 8–10% was explained by fat mass or BMI alone, 2.3% by physical exercise, 2.1% by alcohol consumption, 1.1% by smoking, 1.0% by gender and 0.8% by the plasma triglyceride level. These seven factors together accounted for approximately 15% of the variability in plasma NOV levels. Among obese patients enrolled in the study, 15 morbidly obese women underwent laparoscopic Roux-en-Y gastric by pass (RYGB) surgery. Concomitantly with significant (p<0.0005) weight loss (−15% and −20%, 3 and 6 months after surgery, respectively), we observed that plasma NOV concentration was markedly reduced 3 months (−28%; p<0.05) and 6 months (−48%; p<0.0005) after surgery compared to baseline (**Figure 2A**).

**Table 4:** Multiple regression analyses for the association of plasma NOV levels as a dependent variable.

Model 1	β	R^2^ (%)	Cumulated R^2^ (%)	p value
Fat Mass	0.329	8.09	8.09	<0.0001
Physical Exercise	−0.109	2.32	10.41	= 0.009
Alcohol	−.0106	2.05	12.46	= 0.012
Female Gender	−0.115	1.04	13.5	= 0.023
Smoking	−0.102	1.02	14.52	= 0.013

Model 2	β	R^2^ (%)	Cumulated R^2^ (%)	p value
Fat Mass	0.314	8.19	8.19	<0.0001
Physical Exercise	−0.107	2.39	10.58	= 0.009
Alcohol	−0.114	2.26	12.84	= 0.011
Smoking	−.0111	1.14	13.98	= 0.011
LogTG	−0.088	0.78	14.76	= 0.009

Model 3	β	R^2^ (%)	Cumulated R^2^ (%)	p value
BMI	0.302	10.03	10.03	<0.0001
Physical Exercise	−0.089	2.10	12.13	= 0.028
Alcohol	−0.093	2.15	14.28	= 0.020
Smoking	−0.102	1.16	15.44	= 0.008

TG, triglycerides; BMI, body mass index. For each independent determinant of plasma NOV level, the standardized regression coefficient (β), the proportion of the explained variability (R^2^) and cumulated explained variability in plasma NOV level expressed in percentage are given.

### NOV is expressed in human adipose tissue

Using immunohistochemistry, we found that NOV protein was present in both subcutaneous and omental adipose tissue from obese patients (**Figure 2B**). On serial sections stained for NOV, using CD68 (a specific macrophage marker) and perilipin (a specific adipocyte lipid droplet marker), we determined that NOV was not only expressed in adipocytes but also in macrophages, especially those in crown-like structures surrounding the adipocytes. However, not all adipocytes and macrophages were positive for NOV. No staining was observed when using IgG depleted from NOV-specific IgG (flow through, FT) (data not shown).

NOV expression was also assessed in primary cultures of human macrophages and adipocytes. As shown in **Figure 2C–D** NOV expression at mRNA and secreted protein levels could be easily detected in both types of cultures.

### NOV plasma levels and expression in adipose tissue of high fat-fed mice

We next explored the NOV plasma levels in mice (n = 7**–**10) fed a standard diet (SD) or a high fat diet (HFD) for 12 weeks. As shown in Table 5, the average plasma NOV concentration was significantly increased (p<0.01) in HFD-mice compared to SD-mice. Moreover NOV mRNA levels in the visceral adipose tissue was also increased significantly (p<0.01) in HFD-mice compared to SD-mice (**Figure 2E**).

**Table 5:** Plasma NOV levels and fat mass (%) in C57/Bl6 mice on a standard or a high fat diet for 12 weeks.

	Fat mass %	NOV plasma levels (ng/ml)
SD-fed mice	2.99±0.41	3.04±0.14
HFD-fed mice	9.92±0.80***	4.25±0.24**

SD, standard diet; HFD, high fat diet;

** p<0.01,

*** p<0.001 n = 7–10.

Taken together, these data suggest that both in human and in mice there is a link between the expression of NOV and the expansion of the adipose tissue.

## Discussion

In this study, we provide for the first time evidence that BMI and fat mass are major independent determinants of plasma NOV concentration, leading us to propose that NOV expression in adipose tissue could play a critical role in the development of obesity.

A significant gender effect on plasma NOV concentration was found in our cohort with women displaying a higher level of circulating NOV compared to men. Such an effect could be attributed to the higher proportion of fat content in women compared to men. Other mechanisms, such as hormone status, could also contribute to this variation. In the context of the present study, we thus cannot exclude the possibility that, some hormonal regulations might account, at least in part, for inter-individual variability of NOV levels. In particular, it has been previously reported that E2 inhibits the expression of NOV [27]. However, it is relevant to consider that we observed higher circulating levels of NOV in women than in men, suggesting that the specific and significant effect of gender on NOV levels observed in our study might not be related to E2 levels. Interestingly, similar differences were described for plasma leptin. In this latter case, the gender-dependent variation remained significant after normalization with body fat mass, suggesting that other parameters might be responsible for its plasma variation [28], [29].

Beyond the major role of fat mass in the modulation of NOV plasma levels, the question remains whether obesity-associated metabolic alterations related to insulin-resistance are also determinants of NOV concentration. We first examined whether the plasma lipid profile was related to plasma NOV. Interestingly, we observed that plasma triglycerides are positively but weakly associated with the NOV level, however only after adjustment for gender. It is noteworthy that plasma NOV is also linked to physical activity and alcohol consumption, which also modulate plasma triglycerides [30].

Even though high BMI is often associated with impaired glucose tolerance and type 2 diabetes, we did not find any correlation between NOV and glycemia. However, we did observe a positive correlation between NOV and HbA1c which remained significant after adjustment for age, sex, and lipid-lowering therapy. In this context, it is relevant to note that a possible link between NOV and insulin was recently suggested by Shimoyama et al. [17], who reported that NOV expression is reduced in rat aortas after streptozotocin injection and is increased by insulin treatment.

Thus, neither plasma lipids nor glucose seem to be major determinants of plasma NOV in the study population, supporting the assumption that metabolic disorders associated with insulin-resistance do not explain the increased plasma NOV observed in obese patients. However, our study was not designed to compare plasma NOV among control, glucose-intolerant and diabetic patients. Further clinical investigations will be required to establish whether glucose, insulin or insulin-resistance *per se* can modulate plasma NOV. Conversely, we could also consider that NOV may influence lipid and/or glucose homeostasis.

More strikingly, this study reveals for the first time that in our population, plasma NOV concentrations closely correlate with BMI and body fat. This positive correlation is independent of the influence of all other parameters. Interestingly, in patients with an effective weight loss after RYGB, circulating NOV levels drastically decrease 3 and 6 months post-surgery. We also report that NOV is expressed in human adipose tissue from obese patients. Indeed, the NOV transcript and protein are expressed in adipocytes and macrophages. Taken together, these data reveal that NOV expression is closely related to fat mass formation and obesity development. To our knowledge, this is the first study reporting a positive association between NOV and fat excess. Moreover, the positive correlation between circulating NOV and BMI and fat mass could result, at least in part, from the NOV expression in adipose tissue itself. This is also supported by the higher NOV plasma levels and expression in adipose tissue in mice fed with a high fat diet. This represents a remarkable example of a local variation in NOV expression, which could affect its plasma concentration. Indeed, whereas NOV is expressed by several tissues in humans, such as the brain, muscle and most importantly the adrenal cortex [5], our previous studies did not allow us to conclude clearly to what extent a local variation in NOV expression is associated with its plasma level [7].

Which cell types could contribute to NOV expression in adipose tissue? Data drawn from human adipose tissue immunochemistry and from *in vitro* experiments demonstrate that both adipocytes and macrophages can synthesize and secrete NOV. Considering that the adipose tissue of obese subjects is infiltrated with macrophages and contains hypertrophic adipocytes [31], it is tempting to speculate that increased plasma NOV found in obesity is derived from these two cell types. However, their relative contribution remains to be determined.

Several hypotheses could account for NOV increase in obesity. Interestingly, we presently observed a strong positive correlation between NOV and CRP. In this context, several indirect data could link NOV to obesity-related inflammation. NOV expression is regulated by cytokines including TNF-α [3], [19]. It is conceivable that local and systemic expression of NOV could be related to TNF-α expression because this cytokine is locally increased in obesity and mediates the induction of obesity-related factors such as TGF-β, PAI-1 and CCL-2 [32]–[35].

Finally one can wonder about the functions of NOV during obesity. Our group recently reported that NOV can regulate chemokine expression in astrocytes [20] and more importantly, in endothelial [3] and vascular smooth muscle cells. Interestingly, in these latter cells, NOV induces CCL-2 and CCL-5 (unpublished data), which are involved in the immune-inflammatory response of atherosclerosis [36], [37]. It was recently shown that the plasma levels of these two chemokines are increased in obese patients and decreased after Roux-en-Y bariatric surgery [38]. Therefore, our upcoming experiences will aim to evaluate the contribution of NOV to obesity-related inflammation. NOV expression by both adipocytes and macrophages suggest that NOV could participate in an autocrine-paracrine manner to the cross-talk between these cells [39]. NOV-induced chemokine secretion could help immune cell recruitment and in turn modulate adipose tissue and systemic insulin-resistance. Alternatively, recent data suggest that in obesity, extracellular matrix components are essential elements in the remodeling of adipose tissue [40]. As a matricellular multifunctional protein, NOV could also participate in the local remodeling of adipose tissue [2].

In conclusion, our study demonstrates that age, sex and most importantly, BMI influence the NOV level and have to be considered in further studies in which circulating NOV will be used as a biomarker. Finally, we established for the first time a close link between NOV and obesity, very likely through a direct contribution of NOV expression in adipose tissue. These findings represent the first step in unraveling the involvement of NOV in energy homeostasis.
